# Interleukin-8 Producing Hepatocellular Carcinoma with Pyrexia

**DOI:** 10.1155/2009/461492

**Published:** 2009-08-23

**Authors:** Norifumi Harimoto, Ken Shirabe, Tomoyuki Abe, Kiyoshi Kajiyama, Takashi Nagaie, Tomonobu Gion, Yousuke Kuroda, Yoshihiko Maehara

**Affiliations:** ^1^Department of Surgery, Iizuka Hospital, Fukuoka 820-8505, Japan; ^2^Department of Gastroenterological Surgery, Iizuka Hospital, Fukuoka 820-8505, Japan; ^3^Department of Surgery and Science, Graduate School of Medical Sciences, Kyushu University, Fukuoka 812-8582, Japan

## Abstract

We discuss a patient who had poorly differentiated HCC with pyrexia and high CRP in laboratory data, which are not commonly observed in the usual HCC. A 50-year-old man with a history of liver dysfunction was admitted with a chief complaint of a prolonged fever and general fatigue. Preoperative diagnosis was HCC with portal vein tumor thrombus. Posterior segmentectomy of the liver and thrombectomy was performed. Rapid tumor recurrence occurred after surgery, and he died 79 days after the operation. Immunohistochemical stain of HCC in this patient revealed the production of proinflammatory cytokine, interleukin-8 (IL-8). IL-8 production may have contributed to the high fever, high inflammatory reaction, and poor prognosis in this case.

## 1. Introduction

Hepatocellular carcinoma (HCC) is a common malignancy and is now one of the major causes of death in Asian countries [1]. Generally, HCCs are often asymptomatic, but fatigue, abdominal distention and low-grade fever were sometimes observed because of poor liver function. However, fever as a primary symptom in HCC is relatively rare. Additionally, high CRP in laboratory results is also rare. HCC with pyrexia has been reported only in a few studies [2–4] and the cause of pyrexia has remained unknown. 

 Recently, increased production of proinflammatory cytokines and chemokines, such as interleukin-1 *α* (IL-1*α*), interleukin-1 *β* (IL-1*β*), interleukin-6(IL-6), interleukin-8(IL-8), and tumor necrosis factor-*α* (TNF-*α*) have been reported to be febriferous in acute and chronic inflammatory disease [5, 6]. These cytokines are produced by immune cells including various populations of lymphocytes macrophages and other cells. Among the proinflammatory cytokines, IL-8 is a neutrophil chemotactic factor and involved in tumor proliferation and migration, angiogenesis in malignant tumors. This is often accompanied by rapid growth and poor prognosis in malignant tumors [7–13]. 

 We herein discuss a case of HCC accompanied by prolonged spiking fevers, which disappeared after tumor resection. Immunohistochemical stain of HCC in this patient revealed the production of IL-8.

## 2. Case Report

**Figure 1:**
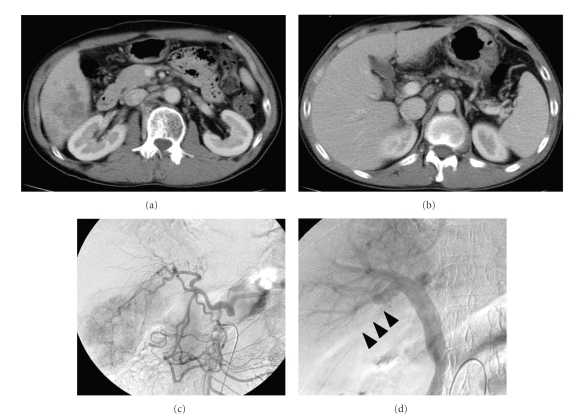
(a) A Computed tomography (CT) showed a peripherally enhanced low density mass 7.5 cm in diameter, which located in segment 6 in the right lobe of the liver. (b) Tumor was accompanied with tumor thrombus to the posterior branch of portal vein. (c) Celiac angiography showed this lesion to be hypervascular. (d) Portal vein tumor thrombus in posterior segment was observed in portal phase of SMA angiography.

A 50-year-old man with a history of liver dysfunction was admitted to Iizuka Hospital with the chief complaint of a prolonged fever and general fatigue in October, 2005. The temperature was over 38°C. Hematological laboratory data on admission were as follows: WBC 9670/ *μ*l, ALP 552 U/l, CRP 16.7 mg/dl, and AFP 11.1 ng/mL. HBs-antigen was positive (Table 1). A Computed tomography (CT) showed a peripherally enhanced low density mass 7.5 cm in diameter, which located in segment 6 in the right lobe of the liver (Figure 1(a)). The tumor was accompanied with tumor thrombus to the posterior branch of portal vein (Figure 1(b)). Celiac angiography showed this lesion was hypervascular (Figure 1(c)) and portal vein tumor thrombus in posterior segment was observed in the portal phase (Figure 1(d)). 

**Figure 2:**
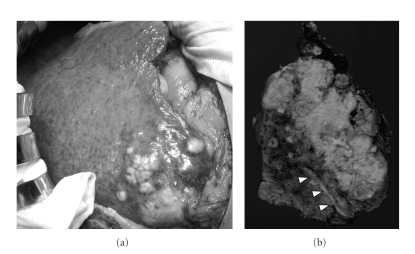
(a) Intraoperative findings revealed tumor was white and intrahepatic metastasis was observed. (b) On cut section, tumor was white and contained coagulation and necrosis. Portal vein tumor thrombus in segment 6 of the liver was observed (arrow heads).

**Figure 3:**
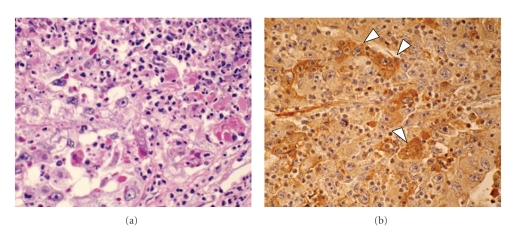
(a) Histological findings revealed poorly differentiated HCC with trabecular pattern. Neutrophil infiltration in the tumor was observed. (b) Immunohistochemically, paraffin section of tumor tissue stained positive for IL-8 (arrow heads).

 Biopsy was not done in this case because of danger of tumor seeding. Patients have chronic hepatitis B and then tumor was hypervascular and accompanied with portal thrombus, which is the feature of HCC, therefore a preoperative diagnosis was made as HCC with portal vein thrombus and nonsteroidal anti-inflammatory drugs were used to reduce the fever. Enough explanation was performed to the patient and informed consent for operation was obtained. Hepatic resection was conducted in November, 2005. The tumor was white and intrahepatic metastasis was observed (Figure 2(a)). A single nodule of disseminated cancer cells was found near the posterior segment on the abdominal wall during the operation. Posterior segmentectomy of the liver and thrombectomy including the dissemination was performed. On the cut section, the tumor was white and contained coagulation and necrosis (Figure 2(b)). Histological findings revealed poorly differentiated HCC with a trabecular pattern. Multiple tumors of intrahepatic metastases and portal vein invasion were observed. Neutrophil infiltration in the tumor was observed (Figure 3(a)). The noncancerous liver tissue showed a moderate chronic inflammatory infiltrate in the fibrous stroma, diagnosed as liver fibrosis. Immunohistochemical stain of IL-8 was performed with paraffin embedded sections of liver tissue using mouse monoclonal antibody at dilutions of 1 : 100 (Assay designs, Ann Arbor, USA). Immunohistochemical staining was described previously [14]. The subsequent reaction was performed by the peroxidase labeled streptavidin-biotin technique using Histofine SAB-PO kit (Nichirei, Tokyo, Japan). IL-8 was observed in the cytoplasm of cancer cells (Figure 3(b)). 

 After the tumor resection, fever disappeared and CRP dropped gradually from 16.7 to 2.7, but the patient had recurrence with multiple liver metastasis and pleuritis carcinomatosa and died on February 2, 2006, 79 days after the operation.

## 3. Discussion

In 1991, Okuda et al. [2] reported five patients with HCC and pyrexia, for an incidence of less than 1%, in Japan. He also mentioned that very poorly differentiated sarcomatoid HCC may frequently appear with pyrexia and leukocytosis mimicking liver abscess. In 1995, Hayashi et al. [3] reported HCC with pyrexia. Histological examination revealed poorly differentiated HCC with sarcomatoid degeneration. Laboratory data revealed high CRP. Nevertheless, in both case reports, the mechanism of a fever was not revealed. In our case, the sarcomatoid change of the cancer cell was not detected, only poorly differentiated HCC. 

 It is well known that cancer cells produce humoral factors and cause the “paraneoplastic syndrome”. Among the clinical symptoms and laboratory data, fever and high CRP, which is not commonly observed in the patients with HCC, is suspected to be due to humoral factors, especially inflammatory cytokine. IL-8 is a proinflammatory cytokine whose principal role in infection and inflammation appears to be the recruitment and activation of circulating and tissue neutrophils to the site of tissue damage. It has been demonstrated that IL-8 is produced by a wide variety of cell types in vitro, including endothelium, monocytes, eosinophils, astrocytes and keratinocytes. In sepsis patients, for example, IL-8 concentrations have been reported as being markedly elevated at diagnosis and remaining high during the course of the illness [15]. Patients with pyrexia is suffering from a high fever and body weight loss. This symptom is induced by many humoral factors, including IL1*β*, IL-6, IL8 and TNF*α*. Zampronio AR reported that IL-8 induced fever by prostaglandin independent mechanism [16]. Additionally, IL-8 was correlated with weight loss in pancreatic cancer [17]. IL-8 resulted in a dose-dependent increase of CRP [18], a decrease in the production of transferring and prealbumin. Therefore, IL-8 is related to pyrexia in parts, not all. 

 In other cancer, Interleukin-8 Producing Malignant Fibrous Histiocytoma with Prolonged Fever was reported [19]. We examined by immunohistochemical staining of IL-8 and tumor tissue stained positive for IL-8 in this case. Akiba et al. [7] reported that a high IL-8 level in HCC had a significantly higher frequency of portal vein invasion and venous invasion and bile duct invasion. In vitro, IL-8 production accelerated the proliferation of endothelial cells. Tachibana et al. [9] reported IL-8 production was enhanced progressively with escalating severity of hepatitis and the development of HCC and the level of IL-8 were significantly increased in patients with advanced HCC with distant metastasis, and then this leads to poor prognosis. Ren et al. reported serum IL-8 level was correlated with tumor size and tumor stage, and then IL-8 level was a significant prognostic factor in terms of disease-free survival and overall survival [13]. Additionally, elevated IL-8 levels in the drainage vein of colorectal cancer are related to the occurrence of hepatic metastasis [10]. This case presented portal vein tumor thrombus, bile duct invasion, intrahepatic metastasis, peritoneal dissemination, early tumor recurrence, rapid tumor regrowth and finally a poor prognosis. In fact, all patients with IL-8 production do not have a high fever, the serum concentration of IL-8 or the degree of neutrophil infiltration will be concerned with pyrexia. 

 In many cancers such as esophageal cancer, gastric cancer and colorectal cancer, serum CRP was known as a prognostic indicator [20–23]. In particular, some reported that serum CRP levels indicated a poor prognosis in HCC patients and portal vein invasion was significantly higher in the serum CRP-positive group [24, 25]. According to the in vitro previous studies, cultured HCC can produce CRP that is regulated in part by proinflammatory cytokines, such as IL-6, IL-8 and TNF-*α* [18, 26, 27]. Among the many proinflammatory cytokines, recent report IL-6 mediates cell cycle arrest in hepatocellular carcinoma through a STAT 3-dependent pathway [28]. Adverse effect may occur according to the type of cytokines. HCC produce proinflammatory cytokines such as IL-8 by autocrine or paracrine, which lead to CRP production and inflammatory cascade or tumor progression. 

 In this report we discussed a patient who had poorly differentiated HCC with pyrexia and high CRP. The patient had surgical resection, but rapid tumor recurrence occurred. IL-8 production was histologically revealed in this case and may have contributed to the high fever, high inflammatory reaction and poor prognosis.

## Figures and Tables

**Table 1:** Laboratory data in this patient. (normal range).

WBC	9670/ *μ*L	(3500–9500)
* *Neut	71.8%	(40–74)
* *Lym	21.5%	(20–74)
Hb	13.2 g/dl	(12.7–17.1)
Plt	34.0 × 10^4^ /*μ*L	(13.8 × 10^4^–33.5 × 10^4^)
PT	77.6%	(86–130)
APTT	41.9 sec	(24.8–36.5)
TP	7.1 g/dl	(6.7–8.3)
Alb	2.6 g/dl	(4.0–5.0)
T.Bil	0.4 mg/dl	(0.3–1.2)
D.Bil	0.1 mg/dl	(0–0.4)
AST	38 U/l	(13–33)
ALT	23 U/l	(6–30)
LDH	330 U/l	(119–229)
ALP	552 U/l	(115–359)
*γ*GTP	79 U/l	(10–47)
ChE	30 U/l	(214–466)
TC	97 mg/dl	(128–219)
TG	48 mg/dl	(30–149)
Amy	111 U/l	(42–132)
BUN	11 mg/dl	(8–22)
Cr	0.6 mg/dl	(0.6–1.1)
FBS	103 mg/dl	(69–109)
CRP	16.7 mg/dl	(<0.2)
ICGR15	11.5%	(<10)
AFP	11.1 ng/mL	(<20)
PIVKA-II	16 mAU/mL	(<40)
CEA	1.1 ng/mL	(<5)
CA19-9	13.5 U/mL	(<37)
HBs-Ag	HBe-Ag (+)	
HBe-Ab	HCV-Ab (−)	
